# Oral antibiotic therapy for the treatment of infective endocarditis: a systematic review

**DOI:** 10.1186/1471-2334-14-140

**Published:** 2014-03-13

**Authors:** Awad Al-Omari, D William Cameron, Craig Lee, Vicente F Corrales-Medina

**Affiliations:** 1 Department of Medicine, Security Forces Hospital, Riyadh, Saudi Arabia; 2 Department of Medicine, The Ottawa Hospital, Ottawa, Ontario, Canada; 3 The Ottawa Hospital Research Institute, Ottawa, Ontario, Canada; 4 The Department of Medicine, University of Ottawa, Ottawa, Ontario, Canada; 5 1053 Carling Ave, ASB 2003, Ottawa, Ontario K1Y 4E9, Canada

**Keywords:** Endocarditis, Treatment, Outcomes, Oral antibiotic therapy

## Abstract

**Background:**

The role of oral antibiotic therapy in treating infective endocarditis (IE) is not well established.

**Methods:**

We searched MEDLINE, EMBASE and Scopus for studies in which oral antibiotic therapy was used for the treatment of IE.

**Results:**

Seven observational studies evaluating the use oral beta-lactams (five), oral ciprofloxacin in combination with rifampin (one), and linezolid (one) for the treatment of IE caused by susceptible bacteria reported cure rates between 77% and 100%. Two other observational studies using aureomycin or sulfonamide, however, had failure rates >75%. One clinical trial comparing oral amoxicillin versus intravenous ceftriaxone for streptococcal IE reported 100% cure in both arms but its reporting had serious methodological limitations. One small clinical trial (n = 85) comparing oral ciprofloxacin and rifampin versus conventional intravenous antibiotic therapy for uncomplicated right-sided *S. aureus* IE in intravenous drug users (IVDUs) reported cure rates of 89% and 90% in each arm, respectively (*P* =0.9); however, drug toxicities were more common in the latter group (62% versus 3%; *P* <0.01). Major limitations of this trial were lack of allocation concealment and blinding at the delivery of the study drug(s) and assessment of outcomes.

**Conclusion:**

Reported cure rates for IE treated with oral antibiotic regimens vary widely. The use of oral ciprofloxacin in combination with rifampin for uncomplicated right-sided *S. aureus* IE in IVDUs is supported by one small clinical trial of relatively good quality and could be considered when conventional IV antibiotic therapy is not possible.

## Background

The hallmark lesion of IE is the endocardial vegetation, a meshwork of platelets, fibrin, bacteria and inflammatory cells in which bacteria proliferate, invade adjacent tissues, and disseminate as septic emboli [1,2]. So long as bacteria are embedded in vegetations, the ability of the immune system to assist with the eradication of these organisms is greatly impaired [1,2]. This characteristic underlies the concept, supported by experimental and clinical observations, that high serum levels of bactericidal antibiotics for prolonged periods of time are needed for curing this infection [1-3]. Before the advent of antibiotic therapy, infective endocarditis (IE) was invariably fatal.

Antibiotics delivered intravenously achieve rapid therapeutic concentrations in blood and perfused tissues, and they are generally regarded as more potent and reliable than their oral counterparts. For these reasons, intravenous (IV) antibiotics are considered the cornerstone of IE treatment [4]. The recommended duration of IV antibiotic therapy for IE varies depending on the characteristics of the infecting organism and the affected endocardial structure but in no instance it is <2 weeks and in most cases it extends beyond 4 weeks [5]. However, there are instances in which the options of effective intravenous antibiotics are limited (patients with multiple allergies, resistant bacteria, etc.) or the maintenance of prolonged intravenous access is not desirable (i.e. active intravenous drug users) or at all feasible (i.e. patient’s inability to maintain intravascular access). In these situations, oral antibiotic therapy can be an attractive and convenient alternative. However, little is known about the value of this strategy in the setting of IE.

Using a systematic approach, this review examines the literature on the efficacy of oral antibiotic therapy in the treatment of IE.

## Methods

Our review protocol conformed to the Preferred Reporting Items for Systematic Reviews and Meta-Analyses (PRISMA) guidelines [6].

### Search strategy

Our systematic search strategy was developed to capture all articles of IE in which oral antibiotic therapy was used. We included articles reporting in English, French, Spanish and Arabic languages. We searched the following databases: MEDLINE (from 1948 to June 1, 2013), EMBASE (from 1947 to June 1, 2013), and Scopus (from 1960 to June 1, 2013). Reference lists of selected papers were also screened for additional articles of interest. The search strategies used are presented in Additional file 1.

### Eligibility criteria

We only included studies of IE in which the duration of antibiotic treatment was >2 weeks and oral antibiotics where the only antibiotics given after 2 weeks of treatment initiation. To be eligible, studies had to *a)* report mortality and clinical cure as their outcomes of interest; *b)* report the microbiology of their IE cases; and *c)* present their data in a way that it allowed for the calculation of outcome rates as a function of the entire study cohort. Studies with focus on culture negative endocarditis were excluded. We also excluded case series (defined as studies with <10 participants) and articles without original data.

### Selection of studies

All titles and abstracts of the citations identified by our literature search were independently screened by two investigators (AA-O and VFC-M). Relevant articles were reviewed in their entirety. Each investigator made a recommendation for inclusion or exclusion of single articles and if discordant, a third investigator solved the discrepancy (CL). When 2 or more articles had overlap of their populations and reported on the same outcomes, only the most inclusive article was considered.

### Data extraction, synthesis and analyses

Using a standardized form, we systematically collected data on the outcomes of interest, the characteristics of the populations studied, whether IE involved the right or left heart valves, and several aspects of the study setting and methodological design. For purposes of this review, cure was defined as both microbiological and clinical resolution of the infection. We used the McMaster University literature appraisal recommendations to evaluate the quality of observational studies [7]; whereas for clinical trials, we used the Consolidated Standards of Reporting Trials (CONSORT) guidelines [8]. We made every effort to calculate pooled incidence rates for the outcomes of interest when feasible.

## Results

**Figure 1:**
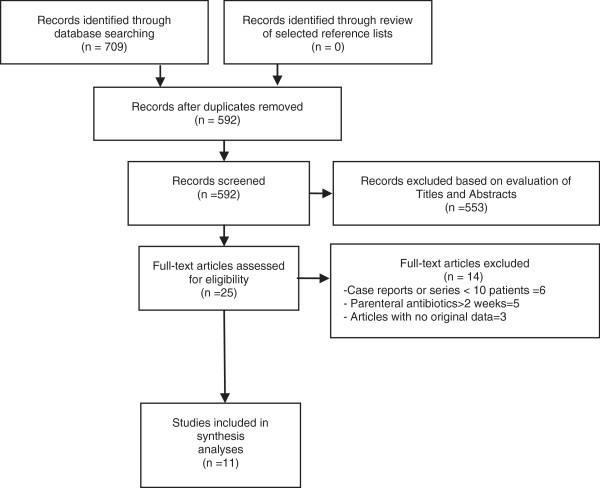
Flow diagram of the process for the selection of articles included in this review.

Of a total of 709 titles retrieved by our search strategy, 25 articles were considered for review based on their title and abstract. Hereafter, 14 more articles were excluded based on exclusion criteria (Additional file 2), leaving 11 studies for the final analyses [9-19] (Figure 1). Of these, 9 were observational [9-17] and 2 were randomized controlled trials (RCTs) [18,19]. Tables 1 and 2 summarize the characteristics of the selected studies.

**Table 1:** Observational studies of oral antibiotic therapy for infective endocarditis

**Reference**	**Cases**	**Design**	**Case definition**	**Microbiology**	**Assessment of antibiotic susceptibility**	**Therapy**	**Cure**
Colli et al, Italy [9]	12 NVIE and 2 PVIE requiring acute valve replacement(all left-sided)	Retrospective. Mean follow-up was 20.8 ± 7 months	By Duke criteria	MRSA (60%)	Yes	IV vancomycin for 5.3 ± 3.4 days followed by oral linezolid for 3 weeks	100%
				*S. viridans* (30%)			
				*Enterococcus sp.* (10%)			
Dworkin et al, USA [10]	13 IVDUs with NVIE (all right-sided with no systemic metastasis)	Prospective. 4-week follow-up	≥2 positive blood cultures AND any of the following: Vegetations on echocardiogram (definite – 3 cases) OR pulmonary infiltrates/effusion or tricuspid insufficiency murmur (probable – 6 cases) OR no other identifiable source for the infection (possible – 1 case)	*S. aureus* (100%)	Yes	IV ciprofloxacin and oral rifampin for 1 week followed by oral ciprofloxacin and oral rifampin for 3 weeks	77%
Chetty et al, South Africa [11]	15 NVIE (right-sided vs. left-sided not specified, all cases were considered uncomplicated)	Prospective. 3-year follow-up	Characteristics clinical features AND any of the following: Positive blood cultures OR vegetations on echocardiogram	*Streptococcus sp*. (60%)	Yes	High dose oral amoxycillin for 6 weeks (47% also received probenecid)	87%
				Culture negative (40%)			
Pinchas et al, Israel [12]	11 NVIE (all left-sided, considered uncomplicated)	Prospective. Follow-up varied from 3 months to 12 years	Fever AND pre-existing valvular heart disease AND multiple positive blood cultures	*S. viridans* (100%)	Yes	High dose oral ampicillin for 6 weeks with probenecid for the first 4 weeks. IM streptomycin for the first 2 weeks	90%
Phillips et al, UK [13]	13 NVIE (right-sided vs. left-sided not specified) – all children	Retrospective. Follow-up varied from 1-15 years	Pre-existing valvular disease AND characteristic clinical features AND positive blood cultures	*S, viridans* (62%) *Staphylococcus sp.* (23%)	Yes	IV therapy for < 2 weeks (92% ≤3 days) followed by oral penicillin V, ampicillin, cloxacillin, flucloxacillin or erythromycin for 6-8 weeks	100%
				Other streptococci or *Enterococcus sp*. (15%)			
Gray et al, UK [14]	13 NVIE (right-sided vs. left-sided not specified)	Retrospective. 3-month follow-up	Not specified	*S. viridans.*(62%)	Yes	Oral ampicillin or propicillin (with or without probenecid) for 6 weeks	92%
				*E. faecalis* (1%)			
				Culture negative (37%)			
Campeau et al, Canada [15]	10 NVIE (right-sided vs. left-sided not specified)	Retrospective. Follow-up varied from 6-30 months	Pre-existing valvular disease AND Characteristic clinical features AND ≥2 positive blood cultures	*S. viridans* (60%)	Yes	Oral phenithicillin for 4-6 weeks (IM streptomycin for the first 2 weeks in 6 cases, concomitant probenecid in 2 cases)	80%
				*E. faecalis* (30%)			
				Anaerobic bacteria (10%)			
Friedberg et al, USA [16]	11 NVIE (right-sided vs. left-sided not specified)	Retrospective. Follow-up not specified	Pre-existing rheumatic valvular disease AND Unexplained fever for ≥2½ weeks	*S. viridans* (55%)	Yes	Oral Aureomycin for 5-8 weeks	36%
				*E. faecalis* (18%)			
				Culture negative (27%)			
Schein et al, USA [17]	81 NVIE (right-side vs. left-sided not specified)	Retrospective. Follow-up varied from 2-8 years	Not specified	*Streptococcus sp.* (94%)	Not specified	Oral sulfonamides (sulfanilamide, sulfapyridine, sulfathiazole or sulfadiazine) for 10 days-14 weeks	10%
				*S. aureus* (1%)			
				*Enterococcus sp.* (1%)			
				*H. influenza* (4%)			

NVIE denotes cases of native valve infective endocarditis. PVIE denotes cases of prosthetic valve infective endocarditis. IV denotes intravenous. IVDUs denotes intravenous drug users. MSSA denotes methicillin-sensitive *S. aureus*. MRSA denotes methicillin-resistant *S. aureus*. CoNS denotes coagulase-negative staphylococcus. GNB denotes gram-negative bacilli. Unless specified otherwise, all cohorts were primarily of adult patients. All reports reported follow-up ≥3 months.

**Table 2:** Clinical trials of oral antibiotic therapy for infective endocarditis

**Reference**	**Cases**	**Design**	**Case definition**	**Microbiology**	**Therapy**	**Results**
Heldman et al, USA [18]	85 IVDUs with NVIE (all right-sided with no systemic metastases), 40 in the oral therapy arm and 45 in the IV therapy arm	Prospective, randomized, open label. 1-month follow-up	- ≥2 positive blood cultures AND any of the following: Valvular vegetations on echocardiogram (definite – 15 cases) OR evidence of pulmonary emboli on chest X-ray or tricuspid insufficiency murmur (probable – 26 cases) OR no other identifiable source for the infection (possible – 44 cases)	MRSA (5%) MSSA (89%) CoNS (6%)	Oral ciprofloxacin and rifampin for 4 weeks vs. IV oxacillin or vancomycin (IV gentamicin for the first 5 days) for 4 weeks	Cure rate: 90% (oral therapy) vs. 91% (IV therapy), *p* = 0.9
						Treatment toxicity: 3% (oral therapy) vs. 62% (IV therapy), *p* < 0.001
Stamboulian et al, Argentine [19]	30 NVIE (all left-sided), 15 in each arm	Prospective, randomized, open label. 3 to 6-motnh follow-up	- ≥2 positive blood cultures AND any of the following: New or changing regurgitant murmur OR predisposing heart disease OR vascular phenomena OR valvular vegetation on echocardiogram	S. viridans (50%)	IV or IM ceftriaxone for 2 weeks followed by high dose oral amoxicillin for 2 weeks vs. IV or IM ceftriaxone for 4 weeks	Cure rate: 100% in both arms. Treatment toxicity not reported
				S. bovis (50%)		


NVIE denotes cases of native valve infective endocarditis. IV denotes intravenous. IM denotes intramuscular. IVDUs denotes intravenous drug users. MSSA denotes methicillin-sensitive *S. aureus*. MRSA denotes methicillin-resistant *S. aureus*. CoNS denotes coagulase-negative staphylococcus. All reports reported follow-up ≥2 months.

### Observational studies

All observational studies involved patients that were hospitalized at the moment of enrolment. Three (33%) of these studies were of prospective design [10-12] and 6 (67%) were retrospective [9,13-17]. Two (22%) studies focused on left- sided IE [9,12], one in right-sided IE (11%) [10], and six (60%) did not specify this information [11,13-17]. One study (11%) involved only intravenous drug users (IVDUs) [10] and one (11%) involved only paediatric cases [13] (Table 1).

#### *Quality assessment*

Only two studies (22%) established a uniform process for patient enrolment [9,10]. One study (11%) relied on modified Duke’s criteria for the diagnosis of IE [9]; two studies (22%) used a combination of suggestive clinical findings, echocardiographic findings and positive blood cultures [10,11]; four studies (44%) made this diagnosis based of the pre-existence of valvular disease and the presence of suggestive clinical findings and/or positive blood cultures [12,13,15,16]; and two studies (22%) did not provide case definitions [14,17]. Only three studies specified inclusion and exclusion criteria [9-11]. No study provided comparative information for eligible patients that were not ultimately enrolled in the study or did comparative analysis of outcomes relative to a control group. All studies gave information of outcomes for all patients enrolled and provided enough information to identify whether the institution in which the investigation was carried out was a referral center or not. The individual quality performance of each of these studies is summarized in Table 3.

**Table 3:** Quality assessment of observational studies of oral antibiotic therapy in infective endocarditis

	**Establishes uniform process for patient enrolment**	**Provides r case-definition for infective endocarditis**	**Provides criteria for inclusion and exclusion**	**Provides comparative information for eligible patients ultimately not enrolled**	**Provides Comparative analysis with a control group**	**All enrolled patients accounted for in the results?**	**It is possible to determine whether the study institutions were referral centers**
**Colli et al**[9]	**√**	**√**	**√**	**X**	**X**	**√**	**√**
**Dworkin et al**[10]	**√**	**√**	**√**	**X**	**X**	**√**	**√**
**Chetty et al**[11]	**X**	**√**	**√**	**X**	**X**	**√**	**√**
**Pinchas et al**[12]	**X**	**√**	**X**	**X**	**X**	**√**	**√**
**Phillips et al**[13]	**X**	**√**	**X**	**X**	**X**	**√**	**√**
**Gray et al**[14]	**X**	**X**	**X**	**X**	**X**	**√**	**√**
**Campeau et al**[15]	**X**	**√**	**X**	**X**	**X**	**√**	**√**
**Friedberg et al**[16]	**X**	**√**	**X**	**X**	**X**	**√**	**√**
**Schein et al**[17]	**X**	**X**	**X**	**X**	**X**	**√**	**√**

#### *Therapy and outcomes*

Four studies (44%) used oral antibiotic therapy for the entire duration of treatment [11,14,16,17], two (22%) used oral therapy along with adjuvant parenteral streptomycin for the first 2 weeks of treatment [12,15], and three (33%) used oral therapy only after an initial short course (<2 weeks) of IV antibiotics [9,10,13]. Oral therapies included beta-lactams (amoxicillin, penicillin V, ampicillin, cloxacillin and dicloxacillin) - with or without probenecid - in five (56%) studies [11-15]; fluoroquinolones (ciprofloxacin) in combination with rifampin in one (16%) [10]; and sulfonamide, aureomycin and linezolid in one study each [9,16,17].

All studies described the microbiological aetiology of their cases. Seven studies (78%) involved patients with infections caused primarily by *Streptococcus sp. *[11-17], while two (22%) included mainly cases of *S. aureus* disease [9,10]. All but one study [17] reported antibiotic susceptibility of the etiologic bacteria.

The follow-up time widely varied among reports (4 weeks to 15 years). Seven studies (78%) reported cure rates between 77-100% [9-15]. Two other studies, however, had failure rates of 90% [17] and 74% [16]. The former of these involved cases of *S. viridians* IE treated with oral sulfonamide [17]; whereas the latter used oral aureomycin to treat *S. viridans* (55%), enterococcus (18%), or culture negative (27%) IE [16] (Table 1).

### Clinical trials

We identified 2 clinical trials. Heldman et al [18] focused on right-sided IE caused by *S. aureus* in IVDUs whereas Stamboulian et al [19] studied left-sided IE caused by *Streptococcus sp.* (*S. viridians* 50% and *S. bovis* 50%) (Table 2).

#### *Quality assessment*

Both studies used a combination of suggestive clinical signs, positive blood cultures and echocardiographic findings in their case definition of IE [18,19]. Both studies also defined eligibility criteria, provided details of the setting and location of the study, and gave a detailed description of the intervention used. Only Heldman et al [18] detailed the processes followed for ascertainment and measurement of clinical outcomes, established adequate sequence generation for randomization, analyzed data on the safety of the intervention, described the participants’ flow in the study, addressed concerns for incomplete outcome data and risk of selective reporting, and provided information on the accessibility to their study protocol, trial registration and the funding for their work. No study complied with concealment or blinding of case allocation at the delivery of the study drug(s) or assessment of outcomes. The individual quality performance of each of these studies is summarized in Table 4.

**Table 4:** Quality assessment of clinical trials of oral antibiotic therapy in infective endocarditis

	**Provides case definition for infective endocarditis**	**Defines eligibility criteria**	**Provides details of the setting and location of the study**	**Provides detailed description of the intervention**	**Details processes for ascertainment and measurement of outcomes**	**Provides justification for sample size**	**Establishes an adequate sequence generation**	**Allocation concealment**	**Blinding (study drug)**	**Blinding (outcomes)**	**Analysis of safety of the intervention**	**Describes participants’ flow**	**Addresses incompleted outcome data**	**Addresses risk of selective reporting**	**Provides information on accessibility to the study protocol, trial registration and funding for the study**
**Heldman et al**[18]	**√**	**√**	**√**	**√**	**√**	**√**	**√**	**X**	**X**	**X**	**√**	**√**	**√**	**√**	**√**
**Stamboulian et al**[19]	**√**	**√**	**√**	**√**	**X**	**X**	**X**	**X**	**X**	**X**	**X**	**N.A.**	**N.A.**	**N.A.**	**X**

N.A. denotes not applicable since Stamboulian et al [20] reported 100% compliance in both treatment arms.

#### *Therapy used and outcomes*

Heldman et al [18] compared 4-week courses of IV vancomycin or oxacillin (with gentamicin for the first five days) vs. oral ciprofloxacin plus rifampin. The cure rates with oral and IV therapy were 89% and 90%, respectively (*P* =0.9). However, drug toxicity was observed in only 1 patient (2.8%) in the oral therapy arm versus 24 (61.5%) in the IV therapy arm (*P* <0.01). Adverse events involved hepatotoxicity (1 patient in the oral therapy arm and in 13 patients in the IV therapy arm), and nephrotoxicity (10 patients in the IV therapy arm).

Stamboulian et al [19] compared a 4-week course of IV ceftriaxone versus 2 weeks of IV ceftriaxone followed by 2 weeks of oral amoxicillin (1 gm four times daily). They reported 100% cure rate in both arms after a follow up of six months (Table 2).

## Discussion

The main findings of our review are: a) Reported cure rates for IE caused by susceptible organisms and treated with appropriate oral antibiotic regimens range between 77-100%; and b) Limited evidence from one small clinical trial suggests that the combination of oral ciprofloxacin and rifampin for the treatment of uncomplicated right-sided IE caused by susceptible strains of *S. aureus* in IVDUs could be as effective as, and produce less adverse events than conventional IV antibiotic regimens.

Previous narrative reviews advocated a limited role for oral antibiotics in the treatment of IE [4]. These opinions, however, were largely based on theoretical considerations and anecdotal experience [4]. In contrast, this study is the first systematic analysis of the existing published evidence on this topic.

The success of antibiotics in controlling bacterial growth and replication is dependent on: a) The susceptibility of the pathogen to the anti-infective that is used; b) The pharmacokinetics of this drug (i.e. whether its bioavailability and distribution allow it to reach the site of infection in sufficient concentration); and, c) Appropriate duration of therapy (especially when the 2 previous criteria are not fully met). Although the first available formulations of antibiotics had unpredictable absorption when given orally, more stable and absorbable compounds soon became available [4]. Oral beta-lactams used in the studies of this review included, among others, ampicillin, amoxicillin, phenithicillin and penicillin V [11-15]. Although the pharmacokinetic profile of oral ampicillin is known to be suboptimal [20], the studies in which this antibiotic was used reported high response rates [12-14]. This is likely explained by the fact that the organisms causing IE in those series were mainly streptococci (which remain highly sensitive to beta-lactams to this date - penicillin MIC ≤0.12), and that large doses of oral ampicillin were used, frequently supplemented with probenecid [12,14]. Oral amoxicillin, on the other hand, has excellent bioavailability (>90%) and low binding to serum proteins (17%), which maximizes its tissue penetration [21]. Typical doses of oral amoxicillin (1 g q8h) produce peak and 6-hour serum concentrations of 16ug/ml and 1.1 ug/ml, respectively [4,22,23]. Further, adding probenecid 1 g to each dose of amoxicillin increases its peak and trough serum concentrations by 30% and 4-fold, respectively [22]. In our review, we found only one observational study reporting 80% cure rate with oral amoxicillin in 15 cases of IE caused mainly by susceptible streptococci, and one poor-quality small clinical trial (n = 30) in which the use of high-dose oral amoxicillin for 2 weeks after 2 initial weeks of IV ceftriaxone resulted in the cure of all patients in the treatment arm [11,19]. Therefore, while pharmacological considerations make oral amoxicillin a plausible alternative for the treatment of IE caused by susceptible bacteria, the clinical evidence supporting this approach is still not robust. However, because streptococci continue to be a leading cause of IE (40% - 60% of native valve endocarditis in the community setting) [1] and oral amoxicillin is inexpensive and widely available, this therapeutic approach should be further investigated in adequately designed clinical trials. Beacause oral penicillin V and phenithicillin also have favourable pharmacokinetic profiles, the same considerations apply to these drugs [24-26].

*S. aureus* is the second most prominent cause of community acquired IE and the leading cause among those who acquired the infection in healthcare settings and among IVDUs [2]. Ciprofloxacin has bactericidal activity against *S. aureus* and a favourable pharmacokinetic profile when given orally (70% bioavailability and serum protein binding rate of 30%), but the emergence of resistance during treatment of *S. aureus* experimental disease is well described [27,28]. Similarly, rifampin is bactericidal against *S.aureus*, has almost complete oral bioavailability, and shows little binding to serum proteins; however, it also has a low threshold for the development of spontaneous resistance during therapy [29]. Although combining both agents has unpredictable effects in their anti-bacterial activity in vitro (i.e. synergistic versus antagonistic), it consistently reduces the development of resistance to either drug [30,31]. We identified one prospective observational study [10] and one randomized clinical trial [18] in which the combination of oral ciprofloxacin and rifampin proved not only effective against uncomplicated *S. aureus* right-sided IE in IVDUs but, in the latter case, it was better tolerated than conventional IV therapy. However, the methodological limitations of these studies (Tables 3 and 4) warrant confirmatory investigations before this approach could be widely adopted. In the meantime, this antibiotic combination regimen should only be used in selected cases for which currently favored IV regimens (including beta-lactams or glycopeptides) are not suitable. Notably, development of resistance to the combination of ciprofloxacin and rifampin has been reported in at least one human case of IE [32]. Newer fluoroquinolones such as levofloxacin and moxifloxacin also have favourable pharmacologic profile when given orally and are bactericidal against *S. aureus,* and in contrast to ciprofloxacin, the development of in-vivo resistance appears rare [27,33,34]. Both levofloxacin and moxifloxacin have also proved effective in animal models of infective endocarditis [35,36] and in anecdotal human cases [37,38]. Therefore, it would also be reasonable to consider the oral formulations of these drugs in future studies for the treatment of this infection. We found that clinical experience with the use of oral anti-staphylococcal penicillins such as cloxacillin and flucloxacillin (used in one report of our review) [13] for the treatment of *S. aureus* IE is very limited and therefore, this approach should only be considered in controlled research settings.

Other oral antibiotics used in the reports identified by our review include linezolid, aureomycin, sulphonamide, and erythromycin [9,10,13,17]. Oral linezolid has excellent pharmacologic profile (bioavailability >99% and serum protein binding rate 30%) and there is a growing body of evidence of its efficacy in serious infections caused by Gram-positive cocci [39]. The promising results with the use of oral linezolid for the treatment IE reported by Colli et al [9] warrant further confirmation in clinical trials. Aureomycin (chlortetracyclin) is an old tetracycline derivative with almost complete GI absorption that is no longer available for clinical use [40]. Aureomycin was only 36% effective in a small series of 11 patients with IE mainly due to *E. faecalis* and strepcocci [16]. However, newer tetracyclines such as minocycline and doxycycline (which also have excellent bioavailability and are active against gram positive organisms) have more recently been proposed as potential oral alternatives for treatment of IE caused by common bacteria [4]. Doxycycline has long been used for treatment of Q fever IE [41,42] but its unpredictable efficacy against *S.aureus* would limit its use in other IE settings. Minocycline, on the other hand, has consistent and reliable activity against gram-positive organisms, including *S. aureus*, and has been effective in animal models of IE caused by this bacterium [43,44]. Anecdotal reports of clinical success with the use oral minocyline in the treatment *S. aureus* IE in humans further support its consideration in future investigations [45,46]. The report by Schein et al [17] describing single sulfa therapy (i.e. sulphonamide) for IE has only historical relevance, as this therapy is no longer available for oral use. Trimethroprim – sulfamethoxazole, however, a more contemporary sulfa-containing drug with excellent oral bioavailability has been shown to be inferior to vancomycin in treating uncomplicated right-side S. aureus IE in IVDU when given intravenously [47]. Finally, experience with the use of oral macrolides such as erythromycin in the treatment of severe infections is very limited and the growing rates of resistance to macrolides among streptococci and staphylococci are a further concern [48-50].

Our review has limitations. It is possible that we missed evidence beyond the boundaries of our search strategies. As mentioned above, a majority of the studies included in our analyses had poor methodological quality. The significant heterogeneity in their study populations and designs prevented us from calculating any meaningful pooled estimates. Comprehensive analyses of drug safety, side effects, and comparative costs were largely lacking. We cannot rule out potential publication bias against studies that found poor effectiveness of oral antibiotic therapy in IE. Finally, since we limited our review to studies in which no parenteral antibiotic was used beyond the two weeks of treatment, the value of oral regimens as step-down therapy for IE beyond this time interval might have not been fully captured.

## Conclusion

In conclusion, oral antibiotics with favourable pharmacokinetic profiles appear effective in treating selected cases of IE caused by susceptible organisms. Because of its favourable pharmacokinetic profile, high-dose oral amoxicillin for the treatment of IE caused by susceptible streptococci is particularly appealing but studies of better quality are needed before further recommendations can be made about this approach in clinical settings. The same considerations apply to the use of oral linezolid in cases of *S. aureus* IE. Oral combination therapy with ciprofloxacin and rifampin appears to be an acceptable alternative for the treatment of uncomplicated right-side endocarditis caused by susceptible strains of *S. aureus* in IVDUs but until adequate clinical trials are available, this approach should be reserved for special situations in which conventional IV antibiotic therapy is not possible or it is undesirable. Ongoing and future investigations should help to better define the role of oral antibiotics in the treatment of IE [51].

## Abbreviations

IE: Infective endocarditis; IV: Intravenous; IVDU: Intravenous drug user; MIC: Minimum inhibitory concentration.

## Competing interests

The authors have no financial or non-financial competing interests to declare.

## Authors’ contributions

Conception of the review: AA-O and VFC-M. Design of the methodology of the review: AA-O, CL, DWC and VFC-M. Critical review and interpretation of selected literature: AA-O, CL, DWC and VFC-M. Writing of the initial draft: AA-O. Critical review and editing of the manuscript: AA-O, CL, DWC, and VFC-M. All authors take responsibility for the integrity of the work. All authors read and approved the final manuscript.

## Pre-publication history

The pre-publication history for this paper can be accessed here:

http://www.biomedcentral.com/1471-2334/14/140/prepub

## Supplementary Material

Additional file 1 Search strategy to identify studies of oral antibiotic therapy in infective endocarditis. Click here for file

Additional file 2 Articles initially considered for analysis but ultimately excluded based on eligibility criteria. Click here for file
