# Supplementary material for: Oral antibiotic therapy for the treatment of infective endocarditis: a systematic review
Source: BMC Infect Dis. 2014 Mar 13;14:140. doi: 10.1186/1471-2334-14-140 (PMC4007569; doi:10.1186/1471-2334-14-140)
Supplement: Additional file 1 — Search strategy to identify studies of oral antibiotic therapy in infective endocarditis. [file 1471-2334-14-140-S1.doc]

**Additional file 1**

**Search strategy to identify studies of oral antibiotic therapy in infective endocarditis**

**1** endocarditis, bacterial/ or endocarditis, subacute bacterial/

**2** bacterial endocarditis.tw.

**3** (staphylococc$ adj1 endocarditis).tw.

**4** infective endocarditis.tw.

**5**  (streptococc$ adj1 endocarditis).tw.

**6** 1 or 2 or 3 or 4 or 5

**7** exp Anti-Bacterial Agents/

**8** (antibacterials or anti-bacterial agent$ or antibacterial agent$).tw.

**9** ((antibiotic or antimicrobial) adj1 (therap$ or treatment$)).tw.

**10** exp Penicillins/ or (penicillin or penicillins).tw.

**11** (amoxicillin or cefaclor or cefadroxil or cephalexin or clinafloxacin or cefdinir or cefditoren or cefixime or cefprozil or ceftibuten or cefuroxime or cephradine or chloramphenicol or cinoxacin or ciprofloxacin or clarithromycin or clindamycin or cloxacillin or dicloxacillin or doxycycline or erythromycin or enoxacin or erithromycin or levofloxacin or lincomycin or linezolid or lomefloxacin or minocycline or moxifloxacin or ofloxacin or oxacillin or rifampin or sparfloxacin or sulfa$ or tetracycline or trimethoprim-sulfa$).af.

**12** or/7-11

**13** (oral or orally).tw.

**14** Administration, Oral/

**15** 13 or 14

**16** 12 and 15

**17** (oral antibiotic$ or oral antimicrobial or oral penicillin$).tw.

**18** 16 or 17

**19** 6 and 18

**20** animals/ not humans/

**21** 19 not 20

**22** case reports.pt.

**23** 21 not 22

**24** 19 not 23
