# Supplementary material for: Oral antibiotic therapy for the treatment of infective endocarditis: a systematic review
Source: BMC Infect Dis. 2014 Mar 13;14:140. doi: 10.1186/1471-2334-14-140 (PMC4007569; doi:10.1186/1471-2334-14-140)
Supplement: Additional file 2 — Articles initially considered for analysis but ultimately excluded based on eligibility criteria. [file 1471-2334-14-140-S2.docx]

**ARTICLES INITIALLY CONSIDERED FOR ANALYSIS BUT ULTIMATELY EXCLUDED BASED ON ELIGIBILITY CRITERIA**

**Case reports or series <10 patients**

Casas Rodriguez R, Lopez Gomez AA, Valiente Frasses RE: **[Subacute bacterial endocarditis treated with oral penicillin: preliminary report]**. *Arch Med Cuba* 1957, **8**(1):1-4.

Santos-Buch CA, Koenig MG, Rogers DE: **Oral treatment of subacute bacterial endocarditis with phenoxymethyl penicillin (penicillin V)**. *N Engl J Med* 1957, **257**(6):249-257.

Guntheroth WG, Cammarano AA, Kirby WM: **Home treatment of infective endocarditis with oral amoxicillin**. *Am J Cardiol* 1985, **55**(9):1231-1232.

Chayakul P, Yipintsoi T: **Intravenous followed by oral antimicrobial therapy for staphylococcal endocarditis**. *J Med Assoc Thai* 1993, **76**(10):559-563.

Garcia Rodriguez JF, Mesias Prego JA, Dominguez Gomez D: **Treatment of endocarditis due to penicillin-susceptible streptococci with a two-week course of ceftriaxone followed by oral amoxicillin**. *Eur J Clin Microbiol Infect Dis* 1992, **11**(10):952-953.

Thandroyen FT, Hallett AF, Asmal AC: **Oral amoxycillin in infective endocarditis**. *S Afr Med J* 1981, **60**(2):45.

**Parenteral antibiotics >2 weeks**

Parker RH, Fossieck BE, Jr.: **Intravenous followed by oral antimicrobial therapy for staphylococcal endocarditis**. *Ann Intern Med* 1980, **93**(6):832-834.

Levine DP, Holley HP, Eiseman I, Willcox P, Tack K: **Clinafloxacin for the treatment of bacterial endocarditis**. *Clin Infect Dis* 2004, **38**(5):620-631.

Quinn EL, Colville JM: **Subacute bacterial endocarditis. Clinical and laboratory observations in 27 consecutive cases treated with penicillin V by mouth**. *N Engl J Med* 1961, **264**:835-842.

Tan JS, Kaplan S, Terhune CA, Jr., Hamburger M: **Successful two-week treatment schedule for penicillin-susceptible streptococcus viridans endocarditis**. *Lancet* 1971, **2**(7738):1340-1343.

Hamburger M, Kaplan S, Walker WF: **Subacute bacterial endocarditis caused by penicillin-sensitive streptococci. Value of oral phenoxymethyl penicillin and intramuscular streptomycin**. *JAMA* 1961, **175**:554-557.

**Articles with no original data**

Gray IR, Tai AR, Wallace JG, Calder JH: **Treatment of Bacterial Endocarditis with Oral Penicillins**. *Postgrad Med J* 1964, **40**:SUPPL:105-111.

Goodman S, Berry RH, Benjamin JE, Schiro HS, Hamburger M: **Subacute bacterial endocarditis treated with oral penicillin**. *Arch Intern Med* 1959, **104**:625-627.

Lindsay AE, Truxal AC: **Oral therapy in bacterial endocarditis**. *Rocky Mt Med J* 1968, **65**(4):33-35.
